# Endovascular Revascularization of Symptomatic Superficial Femoral Artery (SFA) Occlusion After Below-Knee Amputation Despite Absent Distal Runoff

**DOI:** 10.7759/cureus.109305

**Published:** 2026-05-20

**Authors:** Syed Sami N Zafar Ahmed, Babar Ali

**Affiliations:** 1 Medical School, Touro College of Osteopathic Medicine, Great Falls, USA; 2 Cardiology, Adventist HealthCare, Silver Spring, USA

**Keywords:** acute-on-chronic limb ischemia, below knee amputation, catheter directed thrombolysis, collateral circulation, distal runoff, endovascular revascularization, peripheral artery disease, residual limb ischemia, superficial femoral artery occlusion, thrombectomy

## Abstract

Peripheral arterial disease is a major cause of morbidity, with revascularization traditionally aimed at limb preservation and prevention of major amputation. However, management strategies are less clearly defined once amputation has already occurred. In patients with a prior below-knee amputation, the clinical significance of restoring proximal arterial inflow in the absence of a distal limb remains uncertain.

We present a 73-year-old man with a history of peripheral arterial disease who developed subacute, progressive pain in his residual limb along with a stump wound approximately one year after a left below-knee amputation. He had previously been ambulatory with a prosthesis and functionally independent. Examination demonstrated localized tenderness and erythema without signs of infection, and laboratory studies were unremarkable. Vascular imaging revealed complete occlusion of the superficial femoral artery without distal reconstitution, consistent with multilevel arterial occlusion and absence of a conventional distal target for revascularization.

Initial endovascular attempts revealed a high thrombus burden with minimal restoration of flow following angioplasty. A staged approach was therefore pursued, consisting of catheter-directed thrombolysis followed by repeat angiography, mechanical thrombectomy, and adjunctive balloon angioplasty. Despite the absence of distal runoff, successful recanalization of the superficial femoral and popliteal arteries was achieved, with improved perfusion to the residual limb through collateral pathways.

The patient experienced rapid resolution of stump pain following intervention and was able to resume ambulation with his prosthesis prior to discharge. At short-term follow-up, he remained asymptomatic with preserved functional independence.

This case is hypothesis-generating and suggests that, in highly selected post-amputation patients, restoration of proximal arterial inflow may improve collateral-dependent perfusion, relieve ischemic symptoms, and preserve prosthetic function despite absent distal runoff. Because this represents a single subacute ischemic presentation without standardized perfusion metrics or long-term patency data, the findings should be interpreted cautiously within an individualized risk-benefit framework.

## Introduction

Peripheral arterial disease (PAD) continues to represent a substantial source of morbidity, particularly in its most advanced form, chronic limb-threatening ischemia (CLTI). Contemporary management is fundamentally centered on limb preservation, with revascularization serving as the cornerstone of care. Current American College of Cardiology and American Heart Association guidelines emphasize that restoring perfusion is critical not only for preventing major amputation, but also for reducing pain, promoting wound healing, and preserving functional status [1]. In non-emergent settings, these guidelines advocate for multidisciplinary evaluation prior to any amputation, with the explicit goal of identifying revascularization strategies that may salvage the limb and minimize tissue loss [1]. In lower-extremity revascularization, durable patency depends not only on restoring proximal inflow but also on adequate distal outflow, commonly described angiographically as distal runoff. Distal reconstitution refers to reappearance of contrast-filled downstream vessels beyond an occlusion, while collateral-dependent perfusion refers to tissue supply maintained through smaller bypass channels rather than through a continuous native arterial pathway.

This emphasis on early intervention is further supported by clinical trial data. Studies such as BEST-CLI and BASIL-2 have demonstrated that timely revascularization, whether surgical or endovascular, can significantly reduce major adverse limb events and improve amputation-free survival [2,3]. Delays in revascularization have also been associated with worse outcomes, including higher rates of major amputation [4]. However, these trials primarily evaluated patients with salvageable limbs and do not directly address post-amputation ischemia, absent distal runoff, or revascularization performed primarily to preserve residual limb function.

Current guidelines and clinical trials focus almost exclusively on pre-amputation decision-making, with little attention given to patients who later develop symptomatic arterial occlusion in a residual limb [1,5,6]. Following below-knee amputation (BKA), clinical priorities typically shift toward stump healing, rehabilitation, and secondary prevention, rather than further vascular intervention.

This gap raises an important clinical question: in the absence of a distal limb, does restoring proximal arterial inflow provide meaningful clinical and functional benefit? In this report, we describe a patient with prior BKA who developed subacute limb ischemia due to occlusion of the superficial femoral artery and was successfully managed with staged endovascular revascularization despite the absence of distal runoff. This case highlights a scenario not addressed by current guidelines and suggests that revascularization may retain clinical and functional value beyond traditional limb salvage, even without a conventional distal target.

## Case presentation

A 73-year-old man with a history of PAD, hypertension, and chronic obstructive pulmonary disease presented with subacute, progressive pain involving his left residual limb, accompanied by the development of a stump wound. His surgical history was notable for a left BKA performed in 2023 following complications related to infected orthopedic hardware. Prior to this presentation, he was ambulatory with a prosthesis and functionally independent.

Over the course of five to six days, he developed worsening pain localized to the stump, accompanied by erythema, rest pain, and increasing difficulty with weight-bearing. The pain was most prominent with prosthetic weight-bearing and significantly limited prosthesis tolerance, without clinical features strongly suggestive of abscess, acute traumatic injury, or prosthesis-related mechanical breakdown. A stump wound was present without purulent drainage, fluctuance, or exposed bone; detailed wound measurements and exact wound location were not available in the reviewed documentation. Examination of the residual limb demonstrated localized tenderness and erythema without systemic signs of infection. He denied recent trauma, fever, or systemic symptoms. Laboratory evaluation demonstrated a normal leukocyte count (7.9 ×10³/µL) with stable hemoglobin and no significant metabolic derangements, reducing concern for an infectious etiology. Initial evaluation with duplex ultrasonography raised concern for arterial insufficiency, prompting further imaging. Computed tomography angiography (CTA) confirmed complete occlusion of the left superficial femoral artery (SFA). Objective perfusion metrics such as ankle-brachial index, toe pressures, or transcutaneous oxygen measurements were not available, which limited physiologic quantification of ischemia. He was not on chronic anticoagulation at presentation. Formal Wound, Ischemia, and foot Infection (WIfI) staging could not be applied because the patient had previously undergone BKA and therefore lacked an intact foot for standard wound, ischemia, and infection grading. Rutherford classification was also limited by the post-amputation anatomy; however, the patient’s subacute progressive pain, stump wound, impaired prosthetic weight-bearing, and imaging-confirmed arterial occlusion supported clinically significant residual limb ischemia.

**Figure 1:**
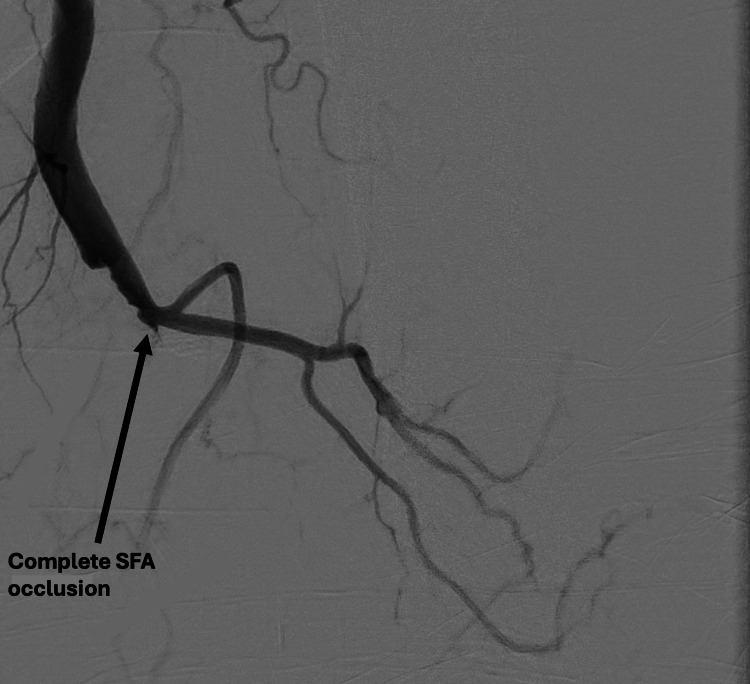
Pre-intervention angiography demonstrating complete occlusion of the mid-superficial femoral artery Pre-intervention anteroposterior angiography of the left lower extremity demonstrating complete occlusion of the mid-superficial femoral artery. The black arrow indicates the site of mid-superficial femoral artery occlusion. Proximal anatomy is oriented superiorly and distal anatomy inferiorly. There is no distal reconstitution into the popliteal or infrapopliteal vessels, with essentially no direct arterial flow reaching the residual limb/stump site. SFA: superficial femoral artery.

The absence of fever, drainage, fluctuance, leukocytosis, or recent trauma made infection, abscess, and mechanical injury less likely, while the temporal progression of pain with weight-bearing difficulty and arterial imaging findings supported an ischemic etiology. Subsequently, the patient was admitted for invasive evaluation and management. Diagnostic angiography demonstrated complete occlusion of the mid-SFA with no distal reconstitution into the popliteal or infrapopliteal vessels (Figure 1). This anatomy represented multilevel occlusion without a conventional distal target for revascularization. Collateral channels were present beyond the level of amputation, suggesting partial compensatory perfusion to the residual limb. Notably, the arterial tree demonstrated relatively limited underlying atherosclerotic disease despite an extensive thrombus burden that appeared disproportionate to the angiographic burden of chronic disease, raising suspicion for an acute thrombotic process, although an embolic source could not be excluded.

Initial attempts to cross the occlusion were technically challenging given the absence of a clear distal target. Percutaneous access was obtained through the right common femoral artery with placement of a 6 Fr sheath. The lesion was initially crossed using a 0.035-inch guidewire and support catheter. Balloon angioplasty of the mid-SFA revealed extensive intraluminal thrombus with persistent total occlusion and minimal restoration of flow.

Given the substantial thrombus burden and multilevel occlusive disease, a staged endovascular strategy was pursued. Catheter-directed thrombolysis was initiated across the occluded segment using alteplase (tPA) infused at 1 mg/hr, for an approximate total alteplase dose of 20 mg over the 20-hour infusion, along with concomitant intravenous heparin per institutional protocol. Thrombolytic infusion was continued for approximately 20 hours with intensive monitoring in the intensive care unit, including serial fibrinogen measurements, activated partial thromboplastin time monitoring, neurovascular examinations, and surveillance for bleeding or compartment syndrome. No major bleeding, neurologic change, or compartment syndrome was documented during thrombolysis or after repeat intervention.

Repeat angiography following thrombolysis demonstrated restoration of antegrade flow within the mid and distal SFA; however, persistent occlusion of the popliteal and infrapopliteal arteries remained, confirming multilevel disease in the absence of a conventional distal target. The occluded popliteal artery and tibial vasculature were subsequently crossed using V14 and Asahi Fielder XT 0.014-inch guidewires with catheter support, with distal wire access achieved toward the peroneal artery near the level of the stump.

Mechanical thrombectomy was then performed using a Penumbra suction thrombectomy system, with multiple passes extracting substantial thrombus from the popliteal artery, tibioperoneal trunk, and peroneal artery. Adjunctive balloon angioplasty was performed using a Sterling SL 3.0 × 150 mm balloon with two inflations to a maximum pressure of 8 atm. Distal embolic protection was not used because there was no suitable distal landing zone or continuous runoff vessel in which a protection device could be safely deployed, limiting the feasibility and expected utility of embolic protection in this anatomy.

**Figure 2:**
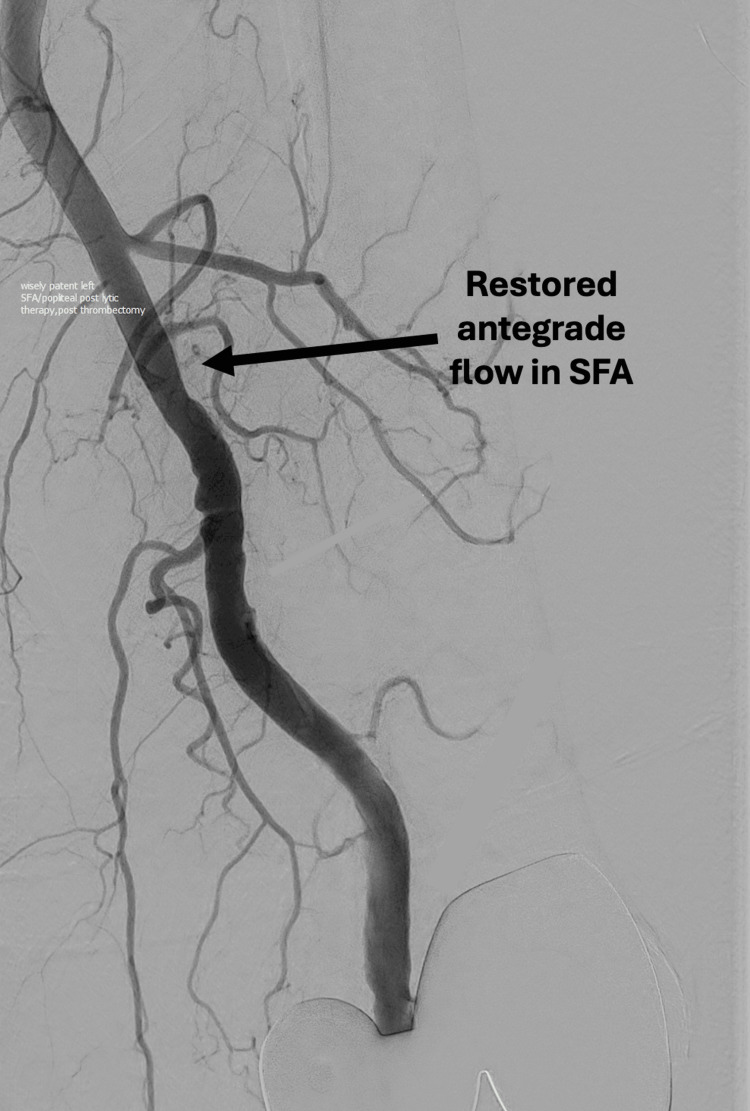
Final post-thrombectomy and post-angioplasty angiography demonstrating restored antegrade flow Final post-thrombectomy and post-angioplasty anteroposterior angiography of the left lower extremity demonstrating restored antegrade flow. The black arrow indicates restored antegrade flow within the superficial femoral artery following catheter-directed thrombolysis, suction thrombectomy, and adjunctive balloon angioplasty. Proximal anatomy is oriented superiorly and distal anatomy inferiorly. Persistent absence of conventional distal runoff is noted, with faint profunda femoris collateral flow supplying the distal popliteal distribution near the residual limb/stump site. SFA: superficial femoral artery.

Final angiographic assessment demonstrated restoration of antegrade flow through the SFA and popliteal artery with improved distal perfusion via collateral pathways, despite residual thrombus within the distal peroneal circulation and absence of conventional distal runoff (Figure 2).

Following intervention, the patient reported rapid and complete resolution of stump pain. He was able to resume ambulation with his prosthesis prior to discharge. The specific antiplatelet regimen and duration were not available in the reviewed documentation. He was referred for outpatient cardiac evaluation, including echocardiography to assess for a potential embolic source. Because the thrombus burden appeared disproportionate to the degree of underlying atherosclerotic disease, an embolic or acute thrombotic component was considered. However, inpatient rhythm monitoring and complete outpatient cardiac evaluation results were not available in the reviewed documentation. Guideline-directed vascular risk reduction, including statin therapy, smoking cessation counseling, and cardiovascular risk-factor optimization, was reinforced at discharge. At short-term follow-up, he remained asymptomatic with preserved functional independence and continued prosthetic ambulation; the exact duration of follow-up was not available in the reviewed documentation. No repeat perfusion testing, duplex surveillance imaging, or transcutaneous oxygen measurements were available at follow-up.

Patient perspective

Before presentation, the patient experienced progressive residual-limb pain that substantially impaired prosthetic weight-bearing and ambulation. He reported worsening discomfort over several days, particularly during prosthesis use, with increasing difficulty performing routine mobility activities because of stump pain and reduced prosthesis tolerance. Following staged endovascular revascularization, he experienced rapid improvement in residual-limb pain and was able to resume prosthesis-assisted ambulation prior to discharge. At short-term follow-up, he remained free of recurrent stump pain and continued to ambulate with preserved functional independence.​​​​​​​ The patient’s clinical timeline is summarized in Table 1.

**Table 1:** Clinical timeline, diagnostic evaluation, intervention, and follow-up. BKA: below-knee amputation; SFA: superficial femoral artery; tPA: tissue plasminogen activator

Time Point	Clinical Events and Findings
2023	Left below-knee amputation (BKA) performed following complications related to infected orthopedic hardware
Post-BKA baseline	Patient recovered well, remained ambulatory with prosthesis, and was functionally independent
5-6 days before presentation	Progressive residual-limb pain, erythema, stump wound development, rest pain, and impaired prosthetic weight-bearing
Presentation/admission	Examination demonstrated localized tenderness and erythema without systemic signs of infection. Leukocyte count was normal (7.9 ×10³/µL). Duplex ultrasonography raised concern for arterial insufficiency
Initial vascular imaging	Computed tomography angiography (CTA) confirmed complete occlusion of the left superficial femoral artery without distal reconstitution
Diagnostic angiography	Angiography demonstrated complete mid-superficial femoral artery occlusion with no distal reconstitution into the popliteal or infrapopliteal vessels, consistent with multilevel occlusive disease without a conventional distal runoff target
Initial intervention	Balloon angioplasty revealed extensive thrombus burden with minimal restoration of flow
Catheter-directed thrombolysis	Alteplase (tPA) infusion at 1 mg/hr, for an approximate total dose of 20 mg over 20 hours, with concomitant intravenous heparin and intensive care monitoring
Repeat intervention	Repeat angiography demonstrated partial restoration of antegrade flow within the superficial femoral artery after thrombolysis. Mechanical thrombectomy using a Penumbra suction thrombectomy system and adjunctive balloon angioplasty were subsequently performed
Final angiographic result	Restoration of antegrade flow through the superficial femoral and popliteal arteries with faint profunda femoris collateral flow supplying the distal popliteal/stump region despite absent conventional distal runoff
Discharge	Rapid resolution of stump pain with return to prosthetic ambulation prior to discharge
Short-term follow-up	Patient remained asymptomatic with preserved functional independence and continued prosthetic ambulation; exact duration of follow-up was not available in the reviewed documentation

## Discussion

This case should be interpreted as a highly selected, symptom- and anatomy-driven intervention rather than evidence for a broad post-amputation revascularization strategy. Contemporary PAD decision-making relies on symptom severity, anatomic feasibility, objective perfusion assessment, expected durability, patient functional goals, and procedural risk. In this patient, formal WIfI staging and objective perfusion metrics were unavailable, and the absence of distal runoff predicted limited durability. The decision to intervene was therefore not based on a standard limb-salvage algorithm, but on progressive residual limb pain, stump wound development, loss of prosthetic function, imaging-confirmed multilevel thrombotic occlusion, and the possibility that restoring proximal inflow could augment collateral-dependent perfusion.

Clinical context and evidence gap

The management of PAD after major limb amputation remains an area of notable clinical uncertainty. While contemporary guidelines provide detailed frameworks for the use of revascularization in preventing limb loss, they offer minimal direction once amputation has already been performed. Current recommendations consistently emphasize pre-amputation evaluation, advocating for multidisciplinary assessment to identify opportunities for revascularization and preserve limb function whenever possible [1,7]. However, these same guidelines provide little practical guidance for patients who later present with symptomatic arterial insufficiency in a residual limb.

Evidence remains limited, as existing guideline statements focus almost exclusively on limb preservation, with limited attention to the management of symptomatic arterial insufficiency after major amputation. As a result, clinicians must often extrapolate from principles developed for intact limbs when managing ischemia in a residual limb [8]. 

In practice, decision-making in this context is inherently individualized. Factors such as vascular anatomy, comorbid conditions, anticipated functional outcomes, and patient goals must all be weighed in the absence of standardized guidance [1,6]. Patients with PAD and prior lower-extremity amputation remain at elevated risk for cardiovascular and limb-related adverse events, underscoring the importance of aggressive vascular risk reduction and optimized medical therapy [6]. Prior reports have described related approaches to post-amputation stump ischemia and inflow compromise. Percutaneous deep venous arterialization at the femoropopliteal segment has previously been reported for management of an unhealed BKA stump ulcer in a patient with superficial femoral artery occlusion and absent runoff [9]. Similarly, retrograde trans-amputation embolectomy of the common, superficial, and deep femoral arteries has been described for inflow revascularization during above-knee amputation [10]. The present case differs from these reports by describing staged arterial endovascular revascularization using catheter-directed thrombolysis, suction thrombectomy, and adjunctive angioplasty for symptomatic residual-limb ischemia after BKA despite absent conventional distal runoff.

Physiologic basis for revascularization in the residual limb

Although direct evidence addressing revascularization after major amputation is limited, established vascular principles provide a coherent physiologic framework for understanding the benefit observed in this case. Tissue viability within the residual limb remains dependent on adequate perfusion, which in turn relies on both primary arterial inflow and the integrity of collateral circulation. Contemporary data demonstrate that while direct revascularization yields the most favorable outcomes, collateral-dependent perfusion can still support tissue viability when supported by sufficient upstream flow [1,11]. This distinction is particularly relevant in the post-amputation setting, where distal arterial targets are absent and perfusion is largely mediated through collateral networks.

Evidence from studies of stump healing reinforces the central role of arterial inflow. Healing of amputation sites has consistently been shown to correlate with the adequacy of perfusion, with proximal arterial patency serving as a key determinant of successful outcomes [12]. More specifically, preservation of inflow through vessels such as the profunda femoris artery has been strongly associated with improved healing, even in the presence of superficial femoral artery occlusion, suggesting that collateral pathways can compensate when adequately supported [12]. These findings underscore that residual limb tissue remains metabolically active and dependent on sustained arterial supply, rather than functionally “disconnected” following amputation. In this setting, restoration of proximal inflow may plausibly improve ischemic symptoms by augmenting collateral-dependent perfusion, although this mechanism was not directly measured in the present case [1]. 

This anatomic framework may help explain the patient’s clinical course. His prior BKA stump had remained viable and functional, suggesting that collateral-supported perfusion, likely through profunda femoris-derived pathways, had previously been sufficient despite underlying chronic femoropopliteal disease [12]. The subsequent thrombus-heavy occlusion may have overwhelmed this previously compensated collateral network, resulting in symptomatic residual-limb ischemia, minimal flow toward the stump site, and impaired prosthetic tolerance.

Technical feasibility of endovascular revascularization

From a technical standpoint, this case also underscores that endovascular intervention may remain feasible even when anatomy appears unfavorable. The anatomy in this case was distinctly challenging: absence of distal reconstitution is generally associated with lower technical success and less durable patency, particularly when occlusion is extensive or distal target vessels are poorly visualized [1]. Distal runoff is critical because it sustains forward flow and maintains vessel patency; when outflow is limited, elevated distal resistance reduces flow and increases the risk of early re-occlusion. Recent data similarly identify lack of target outflow as a predictor of endovascular failure in advanced limb ischemia [13]. These observations make successful lesion crossing and meaningful reperfusion in the present case especially notable.

The procedural sequence used here is well supported by contemporary practice in acute and acute-on-chronic limb ischemia. Current guidelines endorse catheter-directed thrombolysis in patients with salvageable limbs, particularly when thrombotic occlusion is recent and there is an opportunity to restore flow while identifying the underlying culprit lesion [1]. In practice, thrombolysis often serves not as a stand-alone therapy, but as part of a staged strategy that clarifies lesion morphology and facilitates subsequent definitive treatment [1]. This framework aligns closely with the present case, in which a staged strategy was favored due to the absence of a distal target and the high thrombus burden.

The growing role of percutaneous thrombectomy further supports this approach. Contemporary series suggest that mechanical thrombectomy can reduce thrombolytic exposure while achieving acceptable limb salvage and amputation outcomes in infrainguinal ischemia [14]. Likewise, catheter-directed thrombolysis has been shown to be an effective strategy in both acute limb ischemia and acute-on-chronic presentations, with outcomes that appear less dependent on whether the event arises in isolation or on a background of pre-existing arterial disease [15]. In this case, the marked thrombus burden raised concern for an acute thrombotic or embolic component superimposed on chronic vascular pathology, supporting a staged endovascular approach.

Lack of distal reconstitution should be recognized as a marker of procedural difficulty, not necessarily futility. Additionally, when thrombus burden is prominent, a stepwise strategy combining lesion crossing, thrombolysis, thrombectomy, and adjunctive angioplasty may provide a safe and effective route to reperfusion. 

Importantly, the absence of distal embolic protection in this case reflects a procedural reality in no-runoff anatomy, where conventional distal safeguarding strategies are not feasible.​​​​​​​

Reframing the goals of revascularization

This case also invites a broader view of what revascularization is meant to achieve. In CLTI, the traditional objective is clear: preserve a functional limb and avoid major amputation whenever possible [1,16]. That framework remains essential, but it does not fully capture the therapeutic priorities of patients who have already undergone limb loss. In that setting, the relevant clinical outcomes may shift away from limb salvage alone and toward relief of ischemic pain, preservation of stump integrity, maintenance of prosthetic use, and protection of mobility-related quality of life.

That principle becomes especially relevant after a BKA, where functional independence is closely tied to stump viability and prosthetic use. Current guidelines note that successful prosthesis use and return to walking are among the outcomes with the greatest effect on quality of life after amputation [1]. Broader vascular guidance similarly emphasizes that mobility is central to social reintegration and long-term well-being in amputees, and that preservation of the knee joint and adequate tibial length meaningfully improves the likelihood of independent ambulation [5,17]. A painful or poorly perfused residual limb therefore carries consequences that extend well beyond local symptoms; it may directly impair prosthetic tolerance, limit mobility, and diminish quality of life.

Viewed through that lens, the significance of revascularization in this case becomes easier to define. The clinical benefit was not the preservation of a foot, but the restoration of a residual limb capable of supporting prosthetic function without ischemic pain. In other words, the procedural success was meaningful because it preserved mobility. This interpretation is also consistent with broader statements on pain in PAD, which recognize that medical therapy, exercise, and revascularization each contribute to symptom relief and improved function [18]. In selected patients with prior major amputation, the relevant therapeutic goal may therefore shift toward preservation of a viable, pain-free residual limb that supports continued prosthetic use, ambulation, and quality of life.​​​​​​​

Clinical implications and future directions​​​​​​​

From a practical standpoint, this case highlights a scenario for which current PAD frameworks offer little direct guidance. Contemporary recommendations are detailed when the goal is to prevent amputation, but they do not address how to approach a patient with a viable residual limb who later develops symptomatic arterial insufficiency after major amputation [1]. In this setting, the absence of specific guidance does not simply represent a theoretical gap; it creates a real challenge in day-to-day decision-making, where the potential benefits of intervention must be weighed against procedural risk without the support of an established evidence base.

Alternative approaches included conservative medical therapy with analgesia and wound care, pain-directed management alone, or surgical revision to a higher amputation level if tissue compromise progressed. These options were less attractive in this patient because he had been functionally independent with a prosthesis before presentation, had rapidly progressive symptoms, and had imaging evidence of a thrombus-heavy proximal occlusion that appeared technically treatable. Revascularization was therefore pursued as an individualized attempt to relieve ischemic symptoms and preserve residual limb function, rather than as a routine strategy for all no-runoff post-amputation limbs.

The number of patients living with PAD and prior lower-extremity amputation continues to rise, driven in part by the growing burden of diabetes, chronic kidney disease, and advanced vascular disease [6]. At the same time, major amputation remains associated with substantial morbidity and mortality, particularly in older patients and in those with coexisting cardiovascular disease [6]. As more patients survive with residual limbs and depend on them for mobility and independence, clinicians will increasingly encounter situations in which symptom relief, stump preservation, and maintenance of function become central therapeutic concerns.

Durability remains uncertain in this setting. No-runoff anatomy is associated with reduced patency, increased distal resistance, and higher risk of early re-occlusion. The thrombus-heavy nature of the lesion further supports the need for close clinical surveillance, optimization of antithrombotic and vascular risk-reduction therapy, and a low threshold for repeat vascular imaging if symptoms recur. Longer follow-up is needed to determine whether symptom relief and prosthetic function are sustained.

For that reason, a multidisciplinary approach is likely to be essential. Existing PAD guidance already emphasizes that revascularization decisions should be individualized according to symptom burden, functional limitation, anatomy, comorbidities, patient preference, and the expected balance of benefit and risk [1,7]. These principles are even more important in complex post-amputation presentations, where decisions cannot be reduced to a simple limb-salvage algorithm. Input from vascular specialists, interventionalists, rehabilitation teams, wound-care clinicians, and the patient may all be necessary to determine whether intervention is likely to preserve meaningful function [19,20].

More broadly, this case points to the need for a more explicit clinical framework for post-amputation ischemia. Existing staging systems and quality initiatives in PAD have improved the standardization of care for CLTI, but they remain focused on patients with intact limbs [6]. These findings should not be interpreted as support for routine revascularization in all post-amputation no-runoff anatomy, but rather as an example of individualized decision-making in a highly selected patient. Future work should aim to define which post-amputation patients are most likely to benefit from revascularization, what outcomes should be prioritized, and how success should be measured in this setting. Endpoints such as pain relief, stump viability, prosthetic tolerance, ambulatory status, and patient-reported quality of life may prove more relevant than traditional limb-salvage measures alone. Until such data are available, cases such as this one can help inform clinical reasoning in an area where practice currently outpaces evidence.

Limitations

This report has important limitations. Objective perfusion metrics, including ankle-brachial index, toe pressure, skin perfusion pressure, and transcutaneous oxygen measurement, were not available. Formal WIfI staging could not be applied because of prior BKA, and Rutherford classification was limited by the absence of an intact distal limb. Follow-up was limited to short-term symptom resolution and return to prosthetic ambulation, without long-term patency data, completed embolic-source evaluation results, inpatient rhythm-monitoring details, or finalized long-term antithrombotic strategy available in the reviewed documentation. The findings therefore should be considered hypothesis-generating and cannot establish durability, generalizability, or comparative effectiveness against conservative therapy, pain-directed care, or surgical revision.

## Conclusions

This case describes subacute symptomatic ischemia of a residual limb after BKA due to multilevel thrombotic occlusion involving the superficial femoral and popliteal arteries. In this highly selected, previously ambulatory patient, staged endovascular revascularization restored proximal inflow and was followed by resolution of stump pain and return to prosthetic ambulation despite absent conventional distal runoff.

A collateral-augmentation mechanism is plausible but was not directly measured in this case. These findings should therefore be interpreted as hypothesis-generating and should not be generalized to all post-amputation patients with no-runoff anatomy, particularly frail or highly comorbid patients in whom procedural risk may outweigh functional benefit. Future studies should define patient-selection criteria, durability, surveillance strategies, and patient-centered outcomes such as stump viability, prosthesis tolerance, ambulatory status, and quality of life.​
